# Standard radiographic values for the acetabulum in Japanese adolescents: a cross-sectional study

**DOI:** 10.1186/s12891-023-06368-z

**Published:** 2023-04-03

**Authors:** Takahiro Nishimura, Hideaki Watanabe, Naoya Taki, Ichiro Kikkawa, Katsushi Takeshita

**Affiliations:** 1 Department of Orthopaedic Surgery, Jichi Medical University, 3311-1 Yakushiji, Shimotsuke, Tochigi Prefecture 329-0498 Japan; 2 Department of Paediatric Orthopaedics and Orthopaedic Surgery, Jichi Children’s Medical Center, 3311-1 Yakushiji, Shimotsuke, Tochigi Prefecture 329-0498 Japan; 3 Department of Orthopaedic Surgery, Nasu Central Hospital, 1453 Shimoishigami, Otawara, Tochigi Prefecture 324-0036 Japan

**Keywords:** Radiographic parameters, Acetabulum, Standard values, Adolescent, Japanese, Validity, Reliability

## Abstract

**Background:**

Most previous reports of normal acetabular radiographic values focused on adults or elderly people. Recent reports have described premature hip osteoarthritis in adolescents not caused by acetabular dysplasia. In addition, there is a certain failure rate of surgical treatment for young patients with borderline acetabular dysplasia. Accurate indices for treatment of adolescent hips are unclear because standard measurement values of the adolescent acetabulum have not been reported.

**Methods:**

This cross-sectional study involved 552 Japanese adolescents aged 12–18 years who had scoliosis or suspected scoliosis and asymptomatic hips. All persons underwent plain standing anteroposterior whole-spine radiography, and measurements were obtained using the pelvic part of the radiograph. We excluded persons who were unable to correctly perform measurements because of conditions such as pelvic rotation or lateral inclination and persons in whom closure of the triradiate cartilage or closure of the secondary ossification centers of the acetabulum had not yet occurred. In 1101 hips, we measured the lateral center–edge angle (LCEA), Tönnis angle, Sharp angle, acetabular head index (AHI), lateral subluxation (LS), vertical subluxation (VS), and peak-to-edge distance (PED). We evaluated the correlation coefficient and coefficient of determination between each parameter and age, height, body weight, and body mass index (BMI) and assessed the intra- and inter-rater reliability of each radiographic parameter.

**Results:**

Among all hips, the mean of each parameter was as follows: LCEA, 27.9° ± 4.8°; Tönnis angle, 5.0° ± 3.7°; Sharp angle, 44.1° ± 3.1°; AHI, 82.1% ± 5.5%; LS, 5.4 ± 1.4 mm; VS, 0.3 ± 1.2 mm; and PED, 14.0 ± 2.3 mm. The correlation between each parameter and age, height, body weight, and BMI was considerably low. Intra- and inter-rater reliability was moderate or good for almost all parameters.

**Conclusions:**

The values for each radiographic parameter of the acetabulum in this study are considered standard for the adolescent acetabulum without age-related changes. Some parameters differ slightly from the normal values for adults or elderly people in previous reports; thus, we suggest careful evaluation of these parameters for adolescents.

## Background

Acetabular dysplasia (AD) is a cause of hip osteoarthritis [1–4]. Especially in Asian populations, and particularly in the Japanese population, AD is reportedly the most common cause of osteoarthritis [5, 6]. However, recent reports have described premature hip osteoarthritis in adolescents not caused by AD [7–9]. Borderline AD is defined by a lateral center–edge angle (LCEA) of 18° or 20°–24° [10–12]. It is a vague group including dysfunction or instability caused by acetabular shallowness with normal joint function [13, 14]. High surgical failure rates for young patients with borderline AD have been reported [15–20], and 94% of these patients have at least one other radiographic feature suggestive of dysplasia [20]. Accurate indices for treatment of adolescent hips are unknown because standard measurement values of the adolescent acetabulum have not been reported. In adults, the LCEA can be affected by sex, age, height, waist circumference, and body mass index (BMI) [21], but these associations in adolescents are unknown.

Image evaluation of the acetabulum is generally performed using plain radiographs [1, 22, 23], often using the anteroposterior pelvic view. However, most standard acetabular parameters have been obtained from adults, especially middle-aged to elderly people [1, 24–30]. Additionally, standard values for the adolescent acetabulum change with age and have not been evaluated for validity and reliability. Therefore, we investigated the standard values of radiographic parameters of the adolescent acetabulum using standing pelvic radiographs and evaluated the validity and reliability of these values.

## Methods

**Fig. 1:**
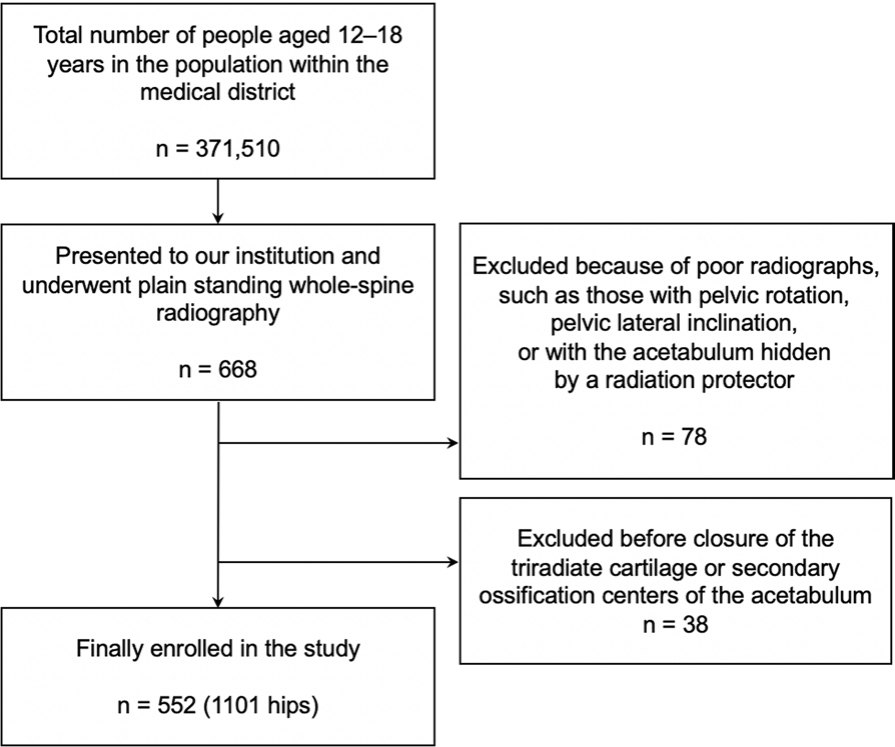
Flow of the persons enrolled in the study

**Fig. 2:**
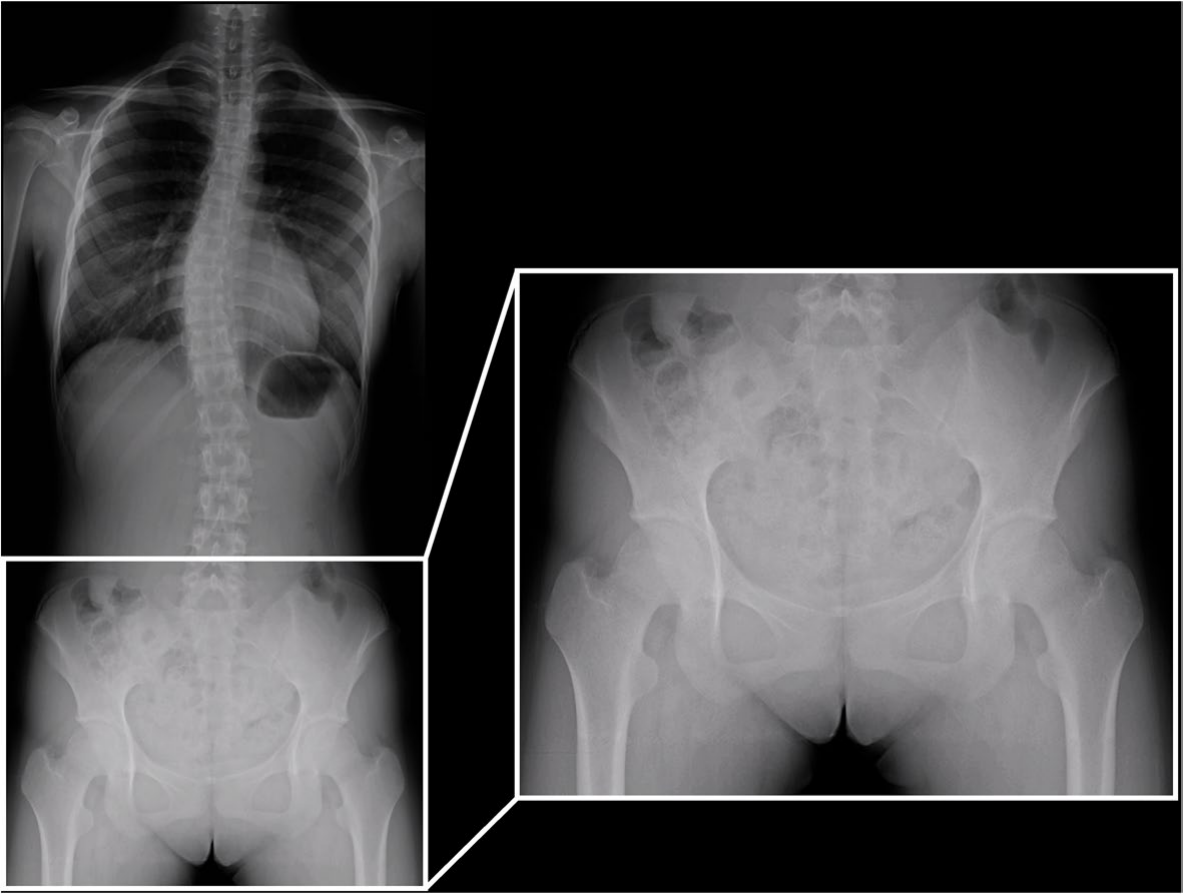
Plain standing whole-spine anteroposterior radiograph and pelvic part of radiograph. With a unified imaging method, it is possible to obtain a stable radiograph with suppressed pelvic rotation and lateral tilt. Measurement was performed using the pelvic part

This study protocol was approved by the ethics review board of our university. Informed consent was obtained from all persons. This cross-sectional study included persons who presented to our outpatient department after being referred for scoliosis or suspected scoliosis from February 2006 to March 2020. Among 371,510 people who comprised the total target age population living in a 1163-km^2^ region around our institution, 668 persons aged 12–18 years were included in the study (Fig. 1). All persons underwent plain standing whole-spine radiography. We enrolled persons with scoliosis or suspected scoliosis because we obtained standing hip radiographs of asymptomatic adolescent hips at the same time as standing whole-spine radiographs. Several parameters were measured using the pelvic part of the radiograph (Fig. 2). We also recorded each person’s age, height, body weight, and BMI to correlate these values with the parameter measurements for validation.

The exclusion criterion was the inability to correctly perform measurements because of conditions such as pelvic rotation, pelvic lateral inclination, or concealment of the acetabulum by a radiation protector. We also excluded persons in whom closure of the triradiate cartilage or closure of the secondary ossification centers of the acetabulum had not yet occurred.

**Fig. 3:**
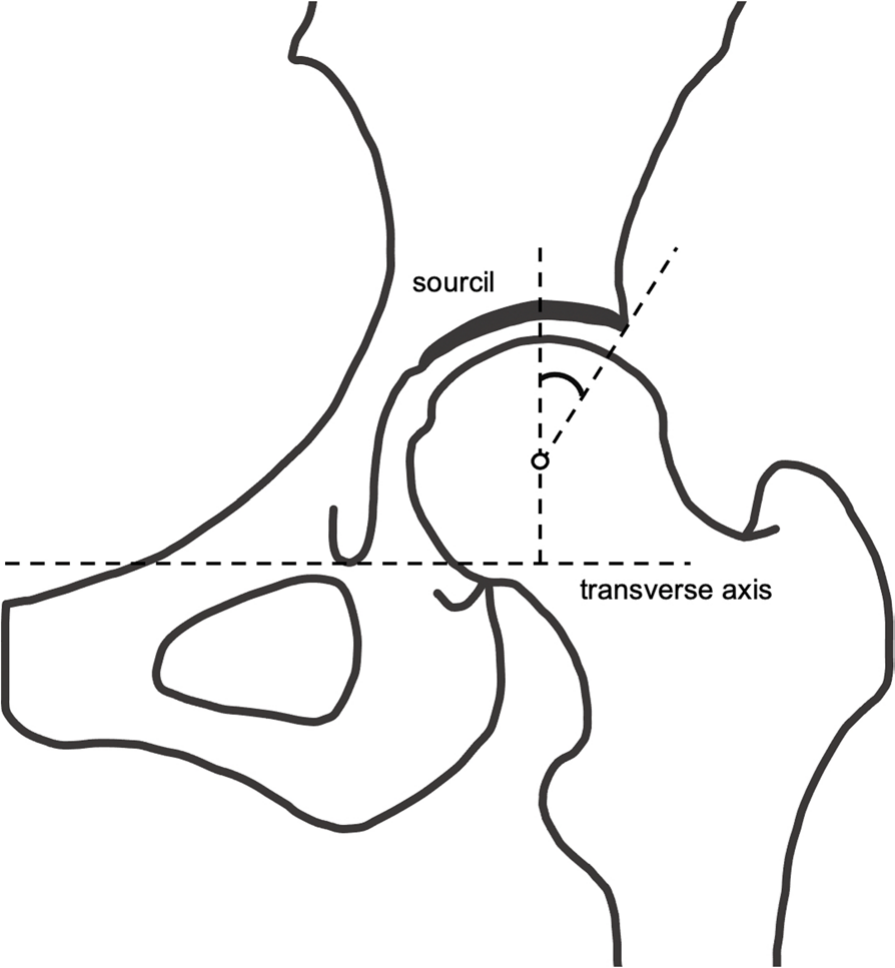
Lateral center–edge angle (LCEA). To determine the LCEA, the first line is drawn through the center of the femoral head perpendicular to the transverse axis of the pelvis. The center of the femoral head is defined using a concentric guide for more accurate measurement. A second line is drawn through the center of the femoral head, passing through the most superolateral point of the sourcil. The sourcil is the weight-bearing zone of the acetabulum, which is seen as osteosclerosis. The LCEA is defined as the intersection of these two lines

**Fig. 4:**
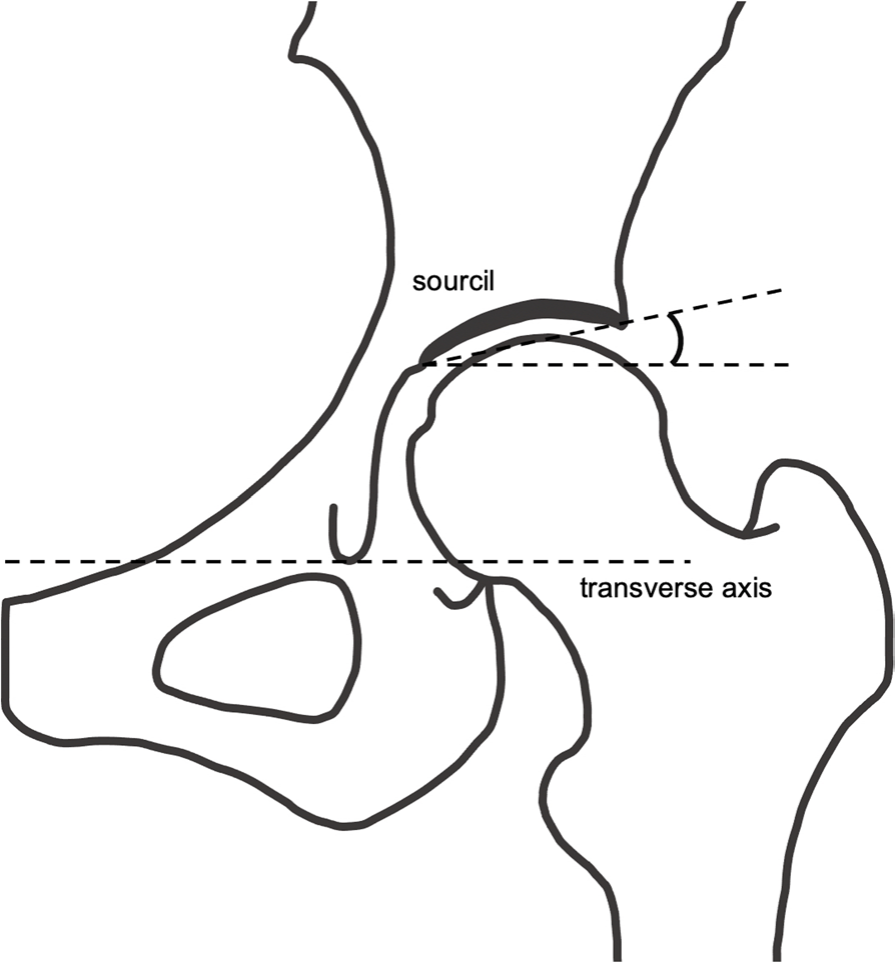
Tönnis angle. To determine the Tönnis angle, a line is drawn connecting the inferior and lateral aspects of the sourcil. A second line is drawn parallel to the transverse axis of the pelvis and through the inferior aspect of the sourcil. The Tönnis angle is created by the intersection of these two lines

**Fig. 5:**
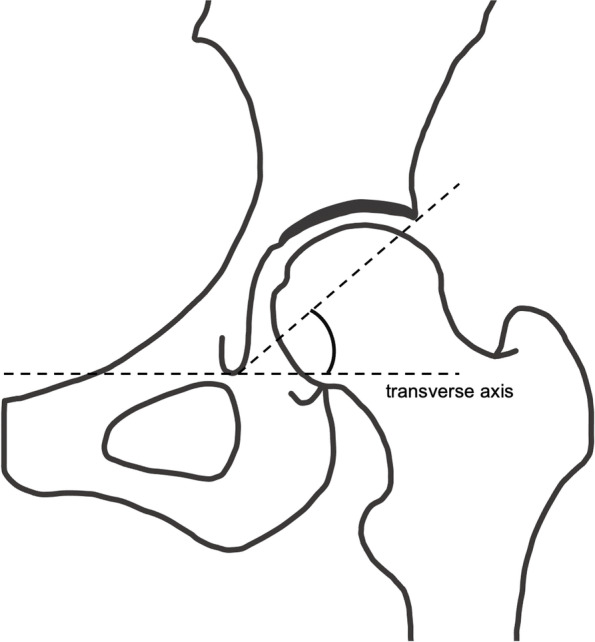
Sharp angle. To determine the Sharp angle, a line is drawn connecting the inferior teardrop point and the lateral rim of the acetabulum. A second line is drawn parallel to the transverse axis of the pelvis through the inferior teardrop point. The Sharp angle is created by the intersection of these two lines

**Fig. 6:**
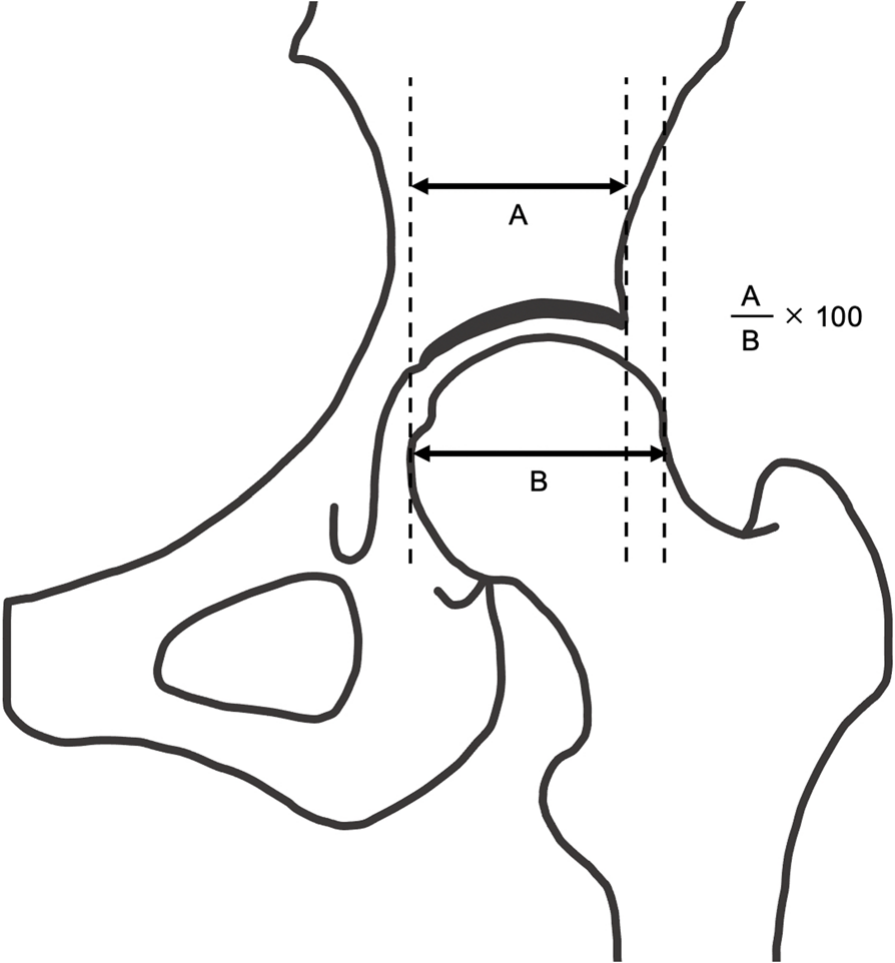
Acetabular head index. The acetabular head index is defined as the percentage calculated by dividing (**A**) the horizontal distance of the femoral head from the medial margin to the edge of the acetabulum by (**B**) the total horizontal width of the femoral head. The formula is A/B × 100

**Fig. 7:**
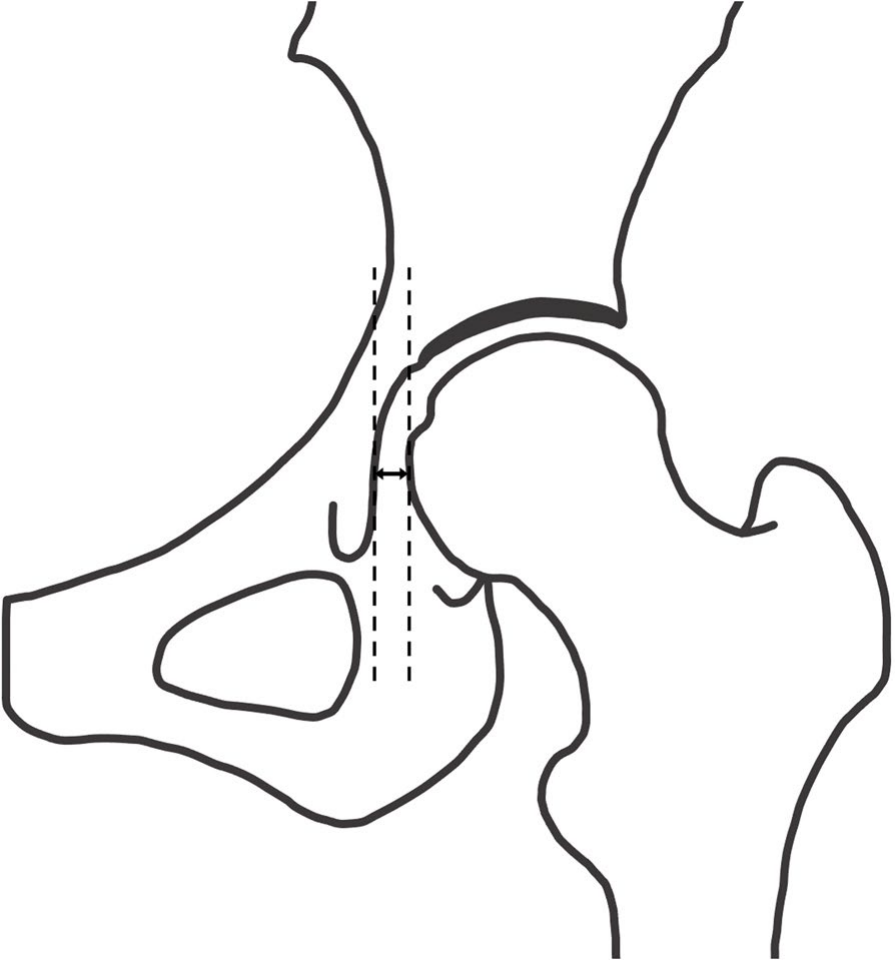
Lateral subluxation (LS). LS is defined as the distance from the medial aspect of the femoral head to the ilioischial line. LS can be evaluated regardless of whether the femoral head is lateralized

**Fig. 8:**
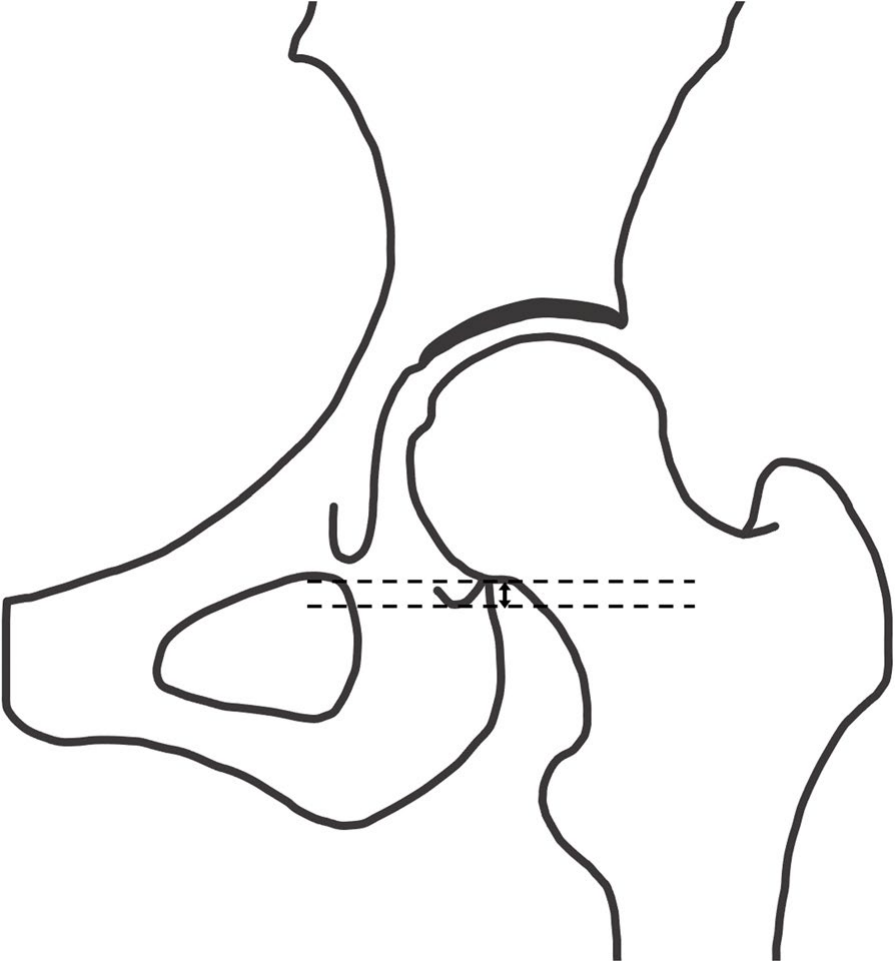
Vertical subluxation. Vertical subluxation is defined as the vertical distance (indicated by the up/down arrow) from the most inferior point of the acetabulum to the most inferior point of the femoral head. In cases in which the most inferior point of the femoral head is above the most inferior point of the acetabulum, the measured value is defined as negative

**Fig. 9:**
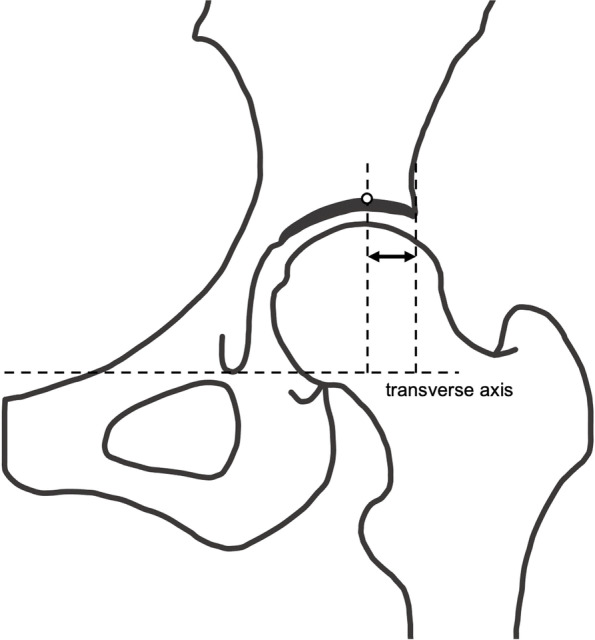
Peak-to-edge distance. A horizontal line is drawn parallel to the transverse axis of the pelvis through the apex of the acetabulum. The apex point is the most proximal point of the dome. The peak-to-edge distance is measured as the horizontal distance from the apex to the acetabular edge (indicated by the left/right arrow)

Radiographs were measured digitally by two orthopedic specialist surgeons. The second measurement was obtained 6 months after the first to evaluate the intra- and inter-rater reliability. The following parameters were measured: LCEA (Fig. 3) [31], Tönnis angle (Fig. 4) [32], Sharp angle (Fig. 5) [33], acetabular head index (AHI) (Fig. 6) [22, 34], lateral subluxation (LS) (Fig. 7) [23], vertical subluxation (VS) (Fig. 8) [1], and peak-to-edge distance (PED) (Fig. 9) [1].

In total, 1101 hips were measured in 552 persons (156 hips of male participants, 945 hips of female participants) (Fig. 1). The mean age was 14.1 years (range, 12–18 years). No persons had hip pain or osteoarthritis.

### Statistical analysis

Statistical analysis was performed using SPSS Version 25 (IBM Corp., Armonk, NY, USA). The mean, standard deviation (SD), and 95% confidence interval (CI) for each parameter were calculated and compared between sexes using the unpaired t-test. The correlation between each parameter and age, height, body weight, and BMI was calculated using Pearson’s product-moment correlation coefficient. Intra- and inter-rater reliability and the 95% CI for each parameter were calculated using intraclass correlation coefficients (< 0.5, poor reliability; 0.5–0.75, moderate reliability; 0.75–0.9, good reliability; > 0.9, excellent reliability) [35]. A *p* value of  ≤ 0.05 was considered statistically significant.

## Results

Tables 1, 2 and 3 summarize the values, correlation coefficients and coefficients of determination (r^2^ values), and intra- and inter-rater reliability of each radiographic parameter of the acetabulum.

**Table 1:** Acetabular parameters in Japanese adolescents measured on plain radiographs

Parameter	All hips (*n* = 1101)	Male (*n* = 156)	Female (*n* = 945)	*p*
LCEA (°)	27.9 ± 4.8	29.8 ± 4.6	27.5 ± 5.0	< 0.001
Tönnis angle (°)	5.0 ± 3.7	3.0 ± 3.1	5.4 ± 3.8	< 0.001
Sharp’s angle (°)	44.1 ± 3.1	43.0 ± 3.1	44.3 ± 3.1	< 0.001
AHI (%)	82.1 ± 5.5	82.9 ± 4.9	81.9 ± 5.7	0.035
LS (mm)	5.4 ± 1.4	6.0 ± 1.2	5.3 ± 1.5	< 0.001
VS (mm)	0.3 ± 1.2	0.9 ± 1.5	0.2 ± 1.1	< 0.001
PED (mm)	14.0 ± 2.3	16.1 ± 2.4	13.7 ± 2.0	< 0.001

Data are presented as mean ± standard deviation

*LCEA* lateral center–edge angle, *AHI* acetabular head index, *LS* lateral subluxation, *VS* vertical subluxation, *PED* peak-to-edge distance

**Table 2:** Correlation coefficient (r) and coefficient of determination (r^2^) for the correlation between each parameter and age, height, body weight, and BMI

Parameter	Correlation coefficient and coefficient determination											
	Age (years)			Height (cm)			Body weight (kg)			BMI (kg/m^2^)		
	r (r^2^)		*p*	r (r^2^)		*p*	r (r^2^)		*p*	r (r^2^)		*p*
LCEA	0.07	(0.00)	0.03	0.13	(0.02)	< 0.001	0.02	(0.00)	0.56	-0.04	(0.00)	0.22
Tönnis angle	-0.11	(0.01)	< 0.001	-0.17	(0.03)	< 0.001	0.04	(0.00)	0.30	0.13	(0.02)	< 0.001
Sharp’s angle	-0.06	(0.00)	0.04	-0.06	(0.00)	0.06	-0.05	(0.00)	0.13	-0.04	(0.00)	0.21
AHI	0.05	(0.00)	0.13	0.07	(0.00)	0.03	0.01	(0.00)	0.83	-0.02	(0.00)	0.59
LS	-0.04	(0.00)	0.25	0.20	(0.04)	< 0.001	0.17	(0.02)	< 0.001	0.06	(0.00)	0.07
VS	-0.01	(0.00)	0.97	0.06	(0.00)	0.08	-0.02	(0.00)	0.50	-0.06	(0.00)	0.07
PED	0.12	(0.01)	< 0.001	0.38	(0.14)	< 0.001	0.15	(0.02)	< 0.001	-0.07	(0.00)	0.06

*BMI* body mass index, *LCEA* lateral center–edge angle, *AHI* acetabular head index, *LS* lateral subluxation, *VS* vertical subluxation, *PED* peak-to-edge distance

**Table 3:** Intra- and inter-rater reliability of acetabular parameters

Parameter	ICC	
	Intra-rater reliability (95% CI)	Inter-rater reliability (95% CI)
LCEA	0.87 (0.86 to 0.89)	0.80 (0.77 to 0.86)
Tönnis angle	0.85 (0.84 to 0.86)	0.71 (0.45 to 0.83)
Sharp’s angle	0.77 (0.75 to 0.79)	0.73 (0.55 to 0.83)
AHI	0.75 (0.73 to 0.77)	0.70 (0.63 to 0.77)
LS	0.66 (0.63 to 0.69)	0.62 (0.51 to 0.71)
VS	0.89 (0.88 to 0.90)	0.57 (0.46 to 0.67)
PED	0.74 (0.72 to 0.76)	0.67 (0.25 to 0.83)

ICC values: < 0.5, poor reliability; 0.5–0.75, moderate reliability; 0.75–0.9, good reliability; > 0.9, excellent reliability

*ICC* intraclass correlation coefficient, *CI* confidence interval, *LCEA* lateral center–edge angle, *AHI* acetabular head index, *LS* lateral subluxation, *VS* vertical subluxation, *PED* peak-to-edge distance

### LCEA

The mean ± SD and 95% CI of the LCEA for all hips, male hips, and female hips was 27.9° ± 4.8° (95% CI, 27.5°–28.2°), 29.8° ± 4.6° (95% CI, 29.1°–30.5°), and 27.5° ± 5.0° (95% CI, 27.3°–27.7°), respectively. There was a significant difference between the sexes (*p* < 0.001). There were significant differences in the correlations between the LCEA and age (*p* = 0.03) and height (*p* < 0.001). However, each r^2^ value was considerably low. The intra- and inter-rater reliability was 0.87 (95% CI, 0.86–0.89) and 0.80 (95% CI, 0.77–0.86), respectively.

### Tönnis angle

The mean ± SD and 95% CI of the Tönnis angle for all hips, male hips, and female hips was 5.0° ± 3.7° (95% CI, 4.8°–5.3°), 3.0° ± 3.1° (95% CI, 2.5°–3.5°), and 5.4° ± 3.8° (95% CI, 5.2°–5.7°), respectively. There was a significant difference between the sexes (*p* < 0.001). There were significant differences in the correlations between the Tönnis angle and age, height, and BMI (*p* < 0.001 for all). However, each r^2^ value was considerably low. The intra- and inter-rater reliability was 0.85 (95% CI, 0.84–0.86) and 0.71 (95% CI, 0.45–0.83), respectively.

### Sharp angle

The mean ± SD and 95% CI of the Sharp angle for all hips, male hips, and female hips was 44.1° ± 3.1° (95% CI, 43.9°–44.3°), 43.0° ± 3.1° (95% CI, 42.5°–43.5°), and 44.3° ± 3.1° (95% CI, 44.1°–44.5°), respectively. There was a significant difference between the sexes (*p* < 0.001). There was a significant difference in the correlations between the Sharp angle and age (*p* = 0.04). However, the r^2^ value was considerably low. The intra- and inter-rater reliability was 0.77 (95% CI, 0.75–0.79) and 0.73 (95% CI, 0.55–0.83), respectively.

### AHI

The mean ± SD and 95% CI of the AHI for all hips, male hips, and female hips was 82.1% ± 5.5% (95% CI, 81.7%–82.5%), 82.9% ± 4.9% (95% CI, 82.2%–83.7%), and 81.9% ± 5.7% (95% CI, 81.6%–82.2%), respectively. There was a significant difference between the sexes (*p* = 0.04). There was a significant difference in the correlations between the AHI and height (*p* = 0.03). However, the r^2^ value was considerably low. The intra- and inter-rater reliability was 0.75 (95% CI, 0.73–0.77) and 0.70 (95% CI, 0.63–0.77), respectively.

### LS

The mean ± SD and 95% CI of LS for all hips, male hips, and female hips was 5.4 ± 1.4 mm (95% CI, 5.3–5.5 mm), 6.0 ± 1.2 mm (95% CI, 5.9–6.2 mm), and 5.3 ± 1.5 mm (95% CI, 5.2–5.4 mm), respectively. There was a significant difference between the sexes (*p* < 0.001). There were significant differences in the correlations between LS and height (*p* < 0.001) and body weight (*p* < 0.001). However, each r^2^ value was considerably low. The intra- and inter-rater reliability was 0.66 (95% CI, 0.63–0.69) and 0.62 (95% CI, 0.51–0.71), respectively.

### VS

The mean ± SD and 95% CI of VS for all hips, male hips, and female hips was 0.3 ± 1.2 mm (95% CI, 0.2–0.4 mm), 0.9 ± 1.5 mm (95% CI, 0.7–1.1 mm), and 0.2 ± 1.1 mm (95% CI, 0.2–0.3 mm), respectively. There was a significant difference between the sexes (*p* < 0.001). There was no significant difference in the correlation between VS and age or body characteristics. The intra- and inter-rater reliability was 0.89 (95% CI, 0.88–0.90) and 0.57 (95% CI, 0.46–0.67), respectively.

### PED

The mean ± SD and 95% CI of the PED for all hips, male hips, and female hips was 14.0 ± 2.3 mm (95% CI, 13.8–14.1 mm), 16.1 ± 2.4 mm (95% CI, 15.7–16.5 mm), and 13.7 ± 2.0 mm (95% CI, 13.6–13.8 mm), respectively. There was a significant difference between the sexes (*p* < 0.001). There were significant differences in the correlations between the PED and age, height, and body weight (*p* < 0.001 for all). However, each r^2^ value was considerably low. The intra- and inter-rater reliability was 0.74 (95% CI, 0.72–0.76) and 0.67 (95% CI, 0.25–0.83), respectively.

## Discussion

The LCEA and AHI indicate the relative acetabular coverage over the femoral head; the Sharp angle, Tönnis angle, and PED reflect the actual acetabular bone morphology; and the LS and VS indicate the degree of femoral head subluxation [1, 23]. Table 4 summarizes the LCEA, Tönnis angle, and Sharp angle in previous representative reports. Notably, however, these reference values were from adults or elderly adults.

**Table 4:** Summary of previous reports of normal radiographic acetabular parameters regarding race, number of samples, age, and reason for the radiograph

Author (Year)	Race	Number of hips	Age (Mean), years	Reason for the radiograph	LCEA (°)		Tönnis angle (°)		Sharp angle (°)	
					M	F	M	F	M	F
Nakamura (1989) [24]	Japanese	254	40 to 69 (54)	Other diseases	32.3	32.1	4.6	5.4	37.3	38.6
Yoshimura (1998) [25]	British	1498	66 to 67 (66)	Urography	36	37				
	Japanese	198	67 to 70 (69)	Urography	31	31				
Han (1998) [26]	Korean	591	22 to 88 (52)	Other diseases	32.6	32.2	5.2	8.0	36.5	37.5
Inoue (2000) [27]	French	401	20 to 79	Urography	37.8	36.9				
	Japanese	782	20 to 79	Urography	35.1	32.8				
Jacobsen (2005) [28]	Danish	4151	M: 23 to 93 (62) F: 22 to 92 (65)	City health study	R: 35.0 L: 34.0	R: 35.0 L: 35.0			R: 37.0 L: 37.0	R: 39.1 L: 38.0
Umer (2006) [29]	Singaporean	522	16 to 99 (60)	Other diseases	30.6	33.5	7.8	7.8		
Im (2010) [30]	Korean	428	17 to 90 (52)	Other diseases	38.0	37.8				

*M* male, *F* female, *R* right, *L* left, *LCEA* lateral center–edge angle

In previous reports, the mean LCEA was 31°–38° for men and 31°–38° for women and was slightly lower for Japanese patients (31°–35° for men and 31°–33° for women) [24–29, 36, 37]. In our study, the mean adolescent LCEA was slightly smaller (29.8° for male and 27.5° for female patients). Recent reports have suggested that analyses based on only the LCEA are limited [14, 38]. However, considering our results and previously reported failure rates of surgical treatment for borderline AD (3–19% for arthroscopic surgery, 9% for osteotomy [15–20]), the diagnosis might be inappropriate. The Tönnis angle in previous reports was 3°–8° [24, 29, 37], with a reference range of 0°–10° [21, 22, 37]. Our findings are similar to these. Previously reported normal values for the Sharp angle were 37°–38° for men, 38°–39° for women, and > 45° in AD [24, 28]. The Sharp angle was slightly larger in the adolescents in the present study. The reported normal mean is 88–93% [28] and ≤ 75% in AD [22]. The Sharp angles in our study were slightly smaller than those in previous reports. Additionally, the previously reported mean LS was 6 mm, and values of < 10 mm were considered normal [1]. However, Clohisy et al. [23] stated that the distance of 10 mm should be considered a general reference as opposed to a strict parameter because magnification errors and variability in patient size can influence this measurement. The normal mean VS in previous reports was 1 mm, and a mean of > 10 mm was considered normal in AD [1]. The VS in our study was 0–1 mm. The LS and VS in our study were similar to those in previous reports. The mean normal PED in previous reports was 16 mm and < 3 mm in AD [1]; the values in our study were slightly smaller.

The adolescent acetabula in our study had a smaller LCEA, larger Sharp angle, and smaller AHI and PED than those in previous reports. These findings indicate that the acetabulum provides slightly less coverage over the acetabular head in adolescents than in adults or elderly people. We considered mainly two factors to explain this difference. The first consideration is age-related changes (e.g., development of osteophytes). In previous reports (Table 4), the survey target populations were adults and elderly adults, and the effects of age should be considered. Lee et al. [39] reported that osteophytes increase the LCEA. Fischer et al. [21] reported that sex, age, height, waist circumference, and BMI could affect the LCEA in adults as evaluated by magnetic resonance imaging. Our study population consisted of adolescents with acetabula that did not change with age; therefore, the results for each parameter in this study are considered the standard values for adolescent acetabula. Second, the bones of the adolescents in this study may have been slightly immature, which may also explain why our values were smaller than those in previous studies. Growth of the acetabulum, or closure of the triradiate cartilage and secondary ossification centers of the acetabulum, occurs around the age of 12–14 years [40–43]. Than et al. [44] also reported that the LCEA gradually increased from 10 to 15 years of age. However, these reports were from outside Japan, and whether the data are applicable to Japanese people is unclear. We targeted persons aged ≥ 12 years and excluded persons in whom closure of the triradiate cartilage or closure of the secondary ossification centers of the acetabulum had not yet occurred to reduce the effects of immaturity as much as possible. Notably, in our study, the correlation between each radiographic parameter and each person’s characteristic (age, height, body weight, and BMI) was considerably low. Therefore, the values for each measurement parameter determined in this study could serve as the standard values for Japanese adolescents aged 12–18 years.

Several studies have evaluated the intra- and inter-rater reliability of acetabular parameters, and the LCEA and Tönnis angle have shown good reliability [37, 39, 45–50]. We found that the intra- and inter-rater reliability was moderate or good for almost all parameters; however, that for the LS was slightly low. We presume that some measurement points may have been difficult to identify in previous studies.

Our study has three main limitations. The first is that whole-spine standing anteroposterior radiographs were used, which differs from previous studies and may have introduced some errors. For a normal standing anteroposterior pelvic radiograph, the film is placed 1.2 m from the X-ray, and the beam center of the X-ray is located cranial to the pubic symphysis. For each person in our study, the film was placed 2 m from the X-ray source, and the center of the X-ray beam was located midway from the xiphoid process to the navel because the lower edge of the eyeball was at the upper edge of the X-ray beam to avoid exposing the crystalline lens. Although the center of the beam was located slightly toward the head, the degree of error in the images was considered small because the incident angle was offset by the long irradiation distance and the large film size. Furthermore, to reduce the error in each case, the persons stood with their patellae forward and locked and their feet shoulder-width apart; they looked straight ahead with their elbows bent and made a fist with each hand in the bilateral supraclavicular fossae. Goldman and Hoover [51] reported that the distance from the beam to the film did not affect the LCEA, Tönnis angle, or Sharp angle. Delagrammaticas et al. [52] reported that a 5° deviation in the beam incidence angle and a 5-cm deviation in the beam center were acceptable. Some reports have indicated that standardization of the orientation and inclination of the pelvis enables more accurate measurement [53, 54]. Furthermore, previous studies on normal values of the acetabulum, which are generally used today, focused on radiographic techniques such as urography and evaluation of other diseases; thus, the data cannot necessarily be obtained from a correct pelvic radiograph [24–30]. We excluded radiographs with pelvic rotation and lateral inclination, minimizing the effect on the radiographic measurements and evaluation. However, the effect of pelvic tilt should be examined in future studies.

The second limitation is that the identification of each measurement point in this study (e.g., lateral rim of the acetabulum, sourcil, or center of the femoral head) did not exactly match those in previous reports, resulting in measurement errors when comparing our results with those in previous reports. Notably, the definitions of measurement points are inconsistent among previous reports [25–27, 36, 55, 56]. Hanson et al. [57] reported that the LCEA depended on whether the measurements were performed using the sourcil or the acetabular margin. Because the measurement method in our study differed from that in previous studies, the standard values must be interpreted with caution.

The final limitation is that all persons were Japanese and had scoliosis or suspected scoliosis. Although there have been no reports of a relationship between scoliosis and AD, the persons with scoliosis or suspected scoliosis in the present study may not be considered normal persons with respect to the acetabular structure. These persons may have had congenital or acquired abnormalities of the bony structure or inclination and rotation of the pelvis. Therefore, to eliminate the influence of scoliosis as much as possible, we fixed each person’s posture for obtaining the radiograph and excluded persons with pelvic rotation or lateral inclination. Additionally, AD is a common cause of hip osteoarthritis in Asians [5, 6]. Furthermore, the study population included a high proportion of female adolescents because they were referred for a scoliosis screening. In previous reports, the average acetabular measurements (especially the LCEA) were smaller in Japanese persons than in persons of other ethnicities [24, 25, 27, 28]. The racial and sex imbalance of the persons may have had a considerable effect on the results of this study.

Our evaluation of the standard values of the adolescent acetabulum, which has no age-related changes, is novel and valuable. Because the correlation between each acetabular measurement and age, height, body weight, and BMI was considerably low in our study, our results could serve as standard values for Japanese adolescents aged 12–18 years. We suggest very careful evaluation of the LCEA, Sharp angle, AHI, and PED when evaluating the adolescent acetabulum. Although careful decision-making is necessary, surgeons should consider surgical treatment for the adolescent acetabulum when the values are outside the range of our findings. We also believe that this will result in fewer surgical failures than when decisions are made based on the reference values for adults.

## Conclusions

We investigated the standard values of radiographic parameters of the acetabulum in 1101 hips of Japanese adolescents aged 12–18 years and evaluated the validity and reliability of these values. The values for each parameter in this study are considered standard for the adolescent acetabulum without age-related changes. Some parameters differ slightly from the standard values for adults or elderly people in previous reports; thus, we suggest careful evaluation of these parameters for adolescents. We believe that our results regarding these standard values will lead to the distinction between “normal” and “pathological” values through future research.

## Data Availability

The datasets used and/or analyzed during the current study are available from the corresponding author on reasonable request.

## Abbreviations

LCEA: Lateral center–edge angle
AHI: Acetabular head index
LS: Lateral subluxation
VS: Vertical subluxation
PED: Peak-to-edge distance
BMI: Body mass index
AD: Acetabular dysplasia
SD: Standard deviation
CI: Confidence interval

## References

[1] Murphy SB, Ganz R, Müller ME (1995). The prognosis in untreated dysplasia of the hip. A study of radiographic factors that predict the outcome. J Bone Joint Surg Am.

[2] Clohisy JC, Dobson MA, Robison JF, Warth LC, Zheng J, Liu SS (2011). Radiographic structural abnormalities associated with premature, natural hip-joint failure. J Bone Joint Surg Am.

[3] Agricola R, Heijboer MP, Roze RH (2013). Pincer deformity does not lead to osteoarthritis of the hip whereas acetabular dysplasia does: acetabular coverage and development of osteoarthritis in a nationwide prospective cohort study (CHECK). Osteoarthritis Cartilage.

[4] Wyles CC, Heidenreich MJ, Jeng J, Larson DR, Trousdale RT, Sierra RJ (2017). The John Charnley Award: redefining the natural history of osteoarthritis in patients with hip dysplasia and impingement. Clin Orthop Relat Res.

[5] Takeyama A, Naito M, Shiramizu K, Kiyama T (2009). Prevalence of femoroacetabular impingement in Asian patients with osteoarthritis of the hip. Int Orthop.

[6] Jingushi S, Ohfuji S, Sofue M (2010). Multiinstitutional epidemiological study regarding osteoarthritis of the hip in Japan. J Orthop Sci.

[7] Baba T, Shitoto K, Kaneko K, Kim S, Maruyama Y (2011). Premature osteoarthritis of the hip in unicyclists: two case reports. Clin J Sport Med.

[8] Nishikino S, Hoshino H, Koyama H, Furuhashi H, Matsuyama Y (2018). Hip arthroscopic surgery after a diagnosis of premature osteoarthritis of the hip in three unicyclists: a case series. J Orthop Case Rep.

[9] Nishimura T, Watanabe H, Taki N, Onuma S, Kikkawa I (2021). Unilateral premature osteoarthritis of the hip with excessive anteversion of the femoral neck developing in the early second decade: two surgical cases. BMC Musculoskelet Disord.

[10] McClincy MP, Wylie JD, Yen YM, Novais EN (2019). mild or borderline hip dysplasia: are we characterizing hips with a lateral center-edge angle between 18° and 25° appropriately?. Am J Sports Med.

[11] Wyatt MC, Beck M (2018). The management of the painful borderline dysplastic hip. J Hip Preserv Surg.

[12] Schmitz MR, Murtha AS, Clohisy JC (2020). Developmental dysplasia of the hip in adolescents and young adults. J Am Acad Orthop Surg.

[13] Bixby SD, Millis MB (2019). The borderline dysplastic hip: when how is it abnormal?. Pediatr Radiol.

[14] Wilkin GP, Ibrahim MM, Smit KM, Beaulé PE (2017). A contemporary definition of hip dysplasia and structural instability: toward a comprehensive classification for acetabular dysplasia. J Arthroplasty.

[15] Chaharbakhshi EO, Perets I, Ashberg L, Mu B, Lenkeit C, Domb BG (2017). Do ligamentum teres tears portend inferior outcomes in patients with borderline dysplasia undergoing hip arthroscopic surgery? A match-controlled study with a minimum 2-year follow-up. Am J Sports Med.

[16] Domb BG, Chaharbakhshi EO, Perets I, Yuen LC, Walsh JP, Ashberg L (2018). Hip arthroscopic surgery with labral preservation and capsular plication in patients with borderline hip dysplasia: minimum 5-year patient-reported outcomes. Am J Sports Med.

[17] Cvetanovich GL, Levy DM, Weber AE (2017). Do patients with borderline dysplasia have inferior outcomes after hip arthroscopic surgery for femoroacetabular impingement compared with patients with normal acetabular coverage?. Am J Sports Med.

[18] Fukui K, Briggs KK, Trindade CA, Philippon MJ (2015). Outcomes after labral repair in patients with femoroacetabular impingement and borderline dysplasia. Arthroscopy.

[19] Hatakeyama A, Utsunomiya H, Nishikino S (2018). Predictors of poor clinical outcome after arthroscopic labral preservation, capsular plication, and cam osteoplasty in the setting of borderline hip dysplasia. Am J Sports Med.

[20] McClincy MP, Wylie JD, Kim YJ, Millis MB, Novais EN (2019). Periacetabular osteotomy improves pain and function in patients with lateral center-edge angle between 18° and 25°, but are these hips really borderline dysplastic?. Clin Orthop Relat Res.

[21] Fischer CS, Kühn JP, Ittermann T (2018). What are the reference values and associated factors for center-edge angle and alpha angle? A population-based study. Clin Orthop Relat Res.

[22] Delaunay S, Dussault RG, Kaplan PA, Alford BA (1997). Radiographic measurements of dysplastic adult hips. Skeletal Radiol.

[23] Clohisy JC, Carlisle JC, Beaulé PE (2008). A systematic approach to the plain radiographic evaluation of the young adult hip. J Bone Joint Surg Am.

[24] Nakamura S, Ninomiya S, Nakamura T (1989). Primary osteoarthritis of the hip joint in Japan. Clin Orthop Relat Res.

[25] Yoshimura N, Campbell L, Hashimoto T (1998). Acetabular dysplasia and hip osteoarthritis in Britain and Japan. Br J Rheumatol.

[26] Han CD, Yoo JH, Lee WS, Choe WS (1998). Radiographic parameters of acetabulum for dysplasia in Korean adults. Yonsei Med J.

[27] Inoue K, Wicart P, Kawasaki T (2000). Prevalence of hip osteoarthritis and acetabular dysplasia in French and Japanese adults. Rheumatology (Oxford).

[28] Jacobsen S, Sonne-Holm S, Søballe K, Gebuhr P, Lund B (2005). Hip dysplasia and osteoarthrosis: a survey of 4151 subjects from the Osteoarthrosis Substudy of the Copenhagen City Heart Study. Acta Orthop.

[29] Umer M, Thambyah A, Tan WT, Das DS (2006). Acetabular morphometry for determining hip dysplasia in the Singaporean population. J Orthop Surg (Hong Kong).

[30] Im GI, Kim JY (2010). Radiological joint space width in the clinically normal hips of a Korean population. Osteoarthritis Cartilage.

[31] Wiberg G (1939). Studies on dysplastic acetabula and congenital subluxation of the hip joint. Acta Orthop Scand.

[32] Tönnis D (1987). Congenital dysplasia and dislocation of the hip in children and adults.

[33] Sharp I (1961). Acetabular dysplasia: the acetabular angle. J Bone Joint Surg [Br].

[34] Heyman C, Herndon C (1950). Legg-Perthes disease: a method for the measurement of the roentgenographic result. J Bone Joint Surg [Am].

[35] Koo TK, Li MY (2016). A guideline of selecting and reporting intraclass correlation coefficients for reliability research. J Chiropr Med.

[36] Lane NE, Nevitt MC, Cooper C, Pressman A, Gore R, Hochberg M (1997). Acetabular dysplasia and osteoarthritis of the hip in elderly white women. Ann Rheum Dis.

[37] Lequesne M, Malghem J, Dion E (2004). The normal hip joint space: variations in width, shape, and architecture on 223 pelvic radiographs. Ann Rheum Dis.

[38] Nepple JJ, Wells J, Ross JR, Bedi A, Schoenecker PL, Clohisy JC (2017). Three patterns of acetabular deficiency are common in young adult patients with acetabular dysplasia. Clin Orthop Relat Res.

[39] Lee YK, Chung CY, Koo KH, Lee KM, Kwon DG, Park MS (2011). Measuring acetabular dysplasia in plain radiographs. Arch Orthop Trauma Surg.

[40] Dimeglio A (2001). Growth in pediatric orthopaedics. J Pediatr Orthop.

[41] Parvaresh KC, Upasani VV, Bomar JD, Pennock AT (2018). Secondary ossification center appearance and closure in the pelvis and proximal femur. J Pediatr Orthop.

[42] Parvaresh KC, Pennock AT, Bomar JD, Wenger DR, Upasani VV (2018). Analysis of acetabular ossification from the triradiate cartilage and secondary centers. J Pediatr Orthop.

[43] Fabricant PD, Hirsch BP, Holmes I (2013). A radiographic study of the ossification of the posterior wall of the acetabulum: implications for the diagnosis of pediatric and adolescent hip disorders. J Bone Joint Surg Am.

[44] Than P, Sillinger T, Kránicz J, Bellyei A (2004). Radiographic parameters of the hip joint from birth to adolescence. Pediatr Radiol.

[45] Chung CY, Park MS, Lee KM (2010). Hip osteoarthritis and risk factors in elderly Korean population. Osteoarthritis Cartilage.

[46] Mast NH, Impellizzeri F, Keller S, Leunig M (2011). Reliability and agreement of measures used in radiographic evaluation of the adult hip. Clin Orthop Relat Res.

[47] Bouttier R, Morvan J, Mazieres B (2013). Reproducibility of radiographic hip measurements in adults. Joint Bone Spine.

[48] Nepple JJ, Martell JM, Kim YJ (2014). Interobserver and intraobserver reliability of the radiographic analysis of femoroacetabular impingement and dysplasia using computer-assisted measurements. Am J Sports Med.

[49] Schottel PC, Park C, Chang A, Knutson Z, Ranawat AS (2014). The role of experience level in radiographic evaluation of femoroacetabular impingement and acetabular dysplasia. J Hip Preserv Surg.

[50] Carreira DS, Emmons BR (2019). The reliability of commonly used radiographic parameters in the evaluation of the pre-arthritic hip: a systematic review. JBJS Rev.

[51] Goldman AH, Hoover KB (2017). Source-to-detector distance and beam center do not affect radiographic measurements of acetabular morphology. Skeletal Radiol.

[52] Delagrammaticas DE, Alvi HM, Kaat AJ, Sullivan RR, Stover MD, Manning DW (2018). Quantitative effect of pelvic position on radiographic assessment of acetabular component position. J Arthroplasty.

[53] Siebenrock KA, Kalbermatten DF, Ganz R (2003). Effect of pelvic tilt on acetabular retroversion: a study of pelves from cadavers. Clin Orthop Relat Res.

[54] Tannast M, Fritsch S, Zheng G, Siebenrock KA, Steppacher SD (2015). Which radiographic hip parameters do not have to be corrected for pelvic rotation and tilt?. Clin Orthop Relat Res.

[55] Engesæter I, Laborie LB, Lehmann TG (2012). Radiological findings for hip dysplasia at skeletal maturity. Validation of digital and manual measurement techniques. Skeletal Radiol.

[56] Kim H, Park JI, Shin DJ, Oh SH, Jeong MY, Yoon PW (2019). Prevalence of radiologic acetabular dysplasia in asymptomatic Asian volunteers. J Hip Preserv Surg.

[57] Hanson JA, Kapron AL, Swenson KM, Maak TG, Peters CL, Aoki SK (2015). Discrepancies in measuring acetabular coverage: revisiting the anterior and lateral center edge angles. J Hip Preserv Surg.

